# Consanguineous Marriages

**Published:** 1867-10

**Authors:** Robert Newman

**Affiliations:** 118 W. Houston Street, New York.


*Consanguineous Marriages.*—We would call attention to the following, and trust it will be responded to by such of our readers as can furnish the required information:

At a late meeting of the "Medical Society of the State of New York," it was resolved, "that a committee be appointed to investigate and report upon the result of consanguineous marriages, &c.," if such marriages come under the observation of the members of the profession, they will confer a favor by answering the following questions, and transmitting such report before November next, to the undersigned, one of the committee appointed:

1. Name (initials) and age of husbands.
2. Nativity.
3. Age when married.
4. Constitution.
5. Health, deformities, peculiar diathesis.
6. Health of his family, hereditary diseases, deformities, &c.
7. Name (initials) and age of wife.
8. Nativity.
9. Age when married.
10. Constitution.
11. Health, deformities, peculiar diathesis.
12. Health of her family, hereditary diseases, deformities, &c.
13. How are the parties related to each other?
14. How long married?
15. How many childern, or sterility?
16. Abortions; cause; how many, and at what period?
17. Children died, at what ages and from what diseases?
18. The constitution, age, and present health of living children, deformities, mental conditions, idiocy, cretenism, deaf, mute, blind, epilepsy, albinism, insane, &c.
19. Remarks and other information.

Address, ROBERT NEWMAN, M.D.,
118 W. Houston Street, New York.

